# Assessing the effects of pharmacists’ perceived organizational support, organizational commitment and turnover intention on provision of medication information at community pharmacies in Lithuania: a structural equation modeling approach

**DOI:** 10.1186/s12913-015-0741-3

**Published:** 2015-03-01

**Authors:** Gvidas Urbonas, Loreta Kubilienė, Raimondas Kubilius, Aušra Urbonienė

**Affiliations:** Department of Social Sciences and Humanities, Medical Academy, Lithuanian University of Health Sciences, Kaunas, Lithuania; Department of Drug Technology and Social Pharmacy, Medical Academy, Lithuanian University of Health Sciences, Kaunas, Lithuania; Department of Cardiology, Medical Academy, Lithuanian University of Health Sciences, Kaunas, Lithuania; Department of Philosophy and Psychology, Kaunas University of Technology, Kaunas, Lithuania

**Keywords:** Perceived organizational support, Organizational commitment, Turnover intention, Provision of medication information

## Abstract

**Background:**

As a member of a pharmacy organization, a pharmacist is not only bound to fulfill his/her professional obligations but is also affected by different personal and organizational factors that may influence his/her behavior and, consequently, the quality of the services he/she provides to patients. The main purpose of the research was to test a hypothesized model of the relationships among several organizational variables, and to investigate whether any of these variables affects the service of provision of medication information at community pharmacies.

**Methods:**

During the survey, pharmacists working at community pharmacies in Lithuania were asked to express their opinions on the community pharmacies at which they worked and to reflect on their actions when providing information on medicines to their patients. The statistical data were analyzed by applying a structural equation modeling technique to test the hypothesized model of the relationships among the variables of Perceived Organizational Support, Organizational Commitment, Turnover Intention, and Provision of Medication Information.

**Results:**

The final model revealed that Organizational Commitment had a positive direct effect on Provision of Medication Information (standardized estimate = 0.27) and a negative direct effect (standardized estimate = −0.66) on Turnover Intention. Organizational Commitment mediated the indirect effects of Perceived Organizational Support on Turnover Intention (standardized estimate = −0.48) and on Provision of Medication Information (standardized estimate = 0.20). Pharmacists’ Turnover Intention had no significant effect on Provision of Medication Information.

**Conclusions:**

Community pharmacies may be viewed as encouraging, to some extent, the service of provision of medication information. Pharmacists who felt higher levels of support from their organizations also expressed, to a certain extent, higher commitment to their organizations by providing more consistent medication information to patients. However, the effect of organizational variables on the variable of Provision of Medication Information appeared to be limited.

## Background

Good Pharmacy Practice guidelines developed by International Pharmaceutical Federation and World Health Organization state that “the mission of pharmacy practice is to contribute to health improvement and help patients to make the best use of their medicines” [1]. To fulfill this mission, pharmacists sell medicines and provide pharmaceutical services and pharmaceutical care, including the service of counseling patients about their medicines. Medication Counseling Behavior Guidelines state the obligation to provide information about indications, dosage regimens, time needed to show an effect of a drug, drug interactions, side effects, and precautions and contraindications, as well as the obligation to provide recommendations on the storage of drugs [2]. It is important to provide patients with all the essential information about medicines to avoid harm due to misuse and so that patients can make informed decisions and receive as many benefits as possible. A European Union directive states that every medicinal drug should be accompanied by a leaflet featuring comprehensive information in a format that is understandable to the patient [3]. However, different studies have shown that the provision of written information in leaflets is a necessary but not sufficient condition for the rational use of medicines. Additional verbal information is required for safe therapy and would support safe and effective use of medicines [4-8]. Studies have also shown that the provision of medication information was not provided to its full extent when dispensing prescriptions [9-12] or non-prescription medicines [13-15]. Responsibility for this provision must be taken by the pharmacist as a healthcare professional and by the pharmacy as a healthcare organization. As a healthcare organization, each community pharmacy has its own organizational culture and climate. As a member of a pharmacy organization, the pharmacist is not only bound to fulfill his/her professional obligations but is also affected by different personal and organizational factors, such as satisfaction with the job, support received from the organization, organizational commitment, and turnover intentions, that may influence his/her behavior and, consequently, the quality of the services he/she provides to patients [16-18].

The organizational support theory developed by Eisenberger and colleagues [19] supposes that each worker has a perception of how the organization cares about employees’ needs and expectations. Multiple studies suggest that perceived organizational support affects employees’ job performance [20], their commitment to the organization, and their tendency to stay with or leave their organizations [21-23]. Organizational commitment as an indicator of organizational effectiveness [24] signifies the employee’s emotional involvement and congruence with his/her organization [25]. Lower levels of organizational commitment may signify lower job performance levels [26,27] and a greater desire to leave the organization [28,29]. Turnover intention is usually viewed as a possible outcome of a negative organizational environment [29] caused by negatively perceived support from the organization and reduced organizational commitment [30]. Increased turnover intention may also lead to negative outcomes, such as reduced job performance [31].

**Figure 1:**
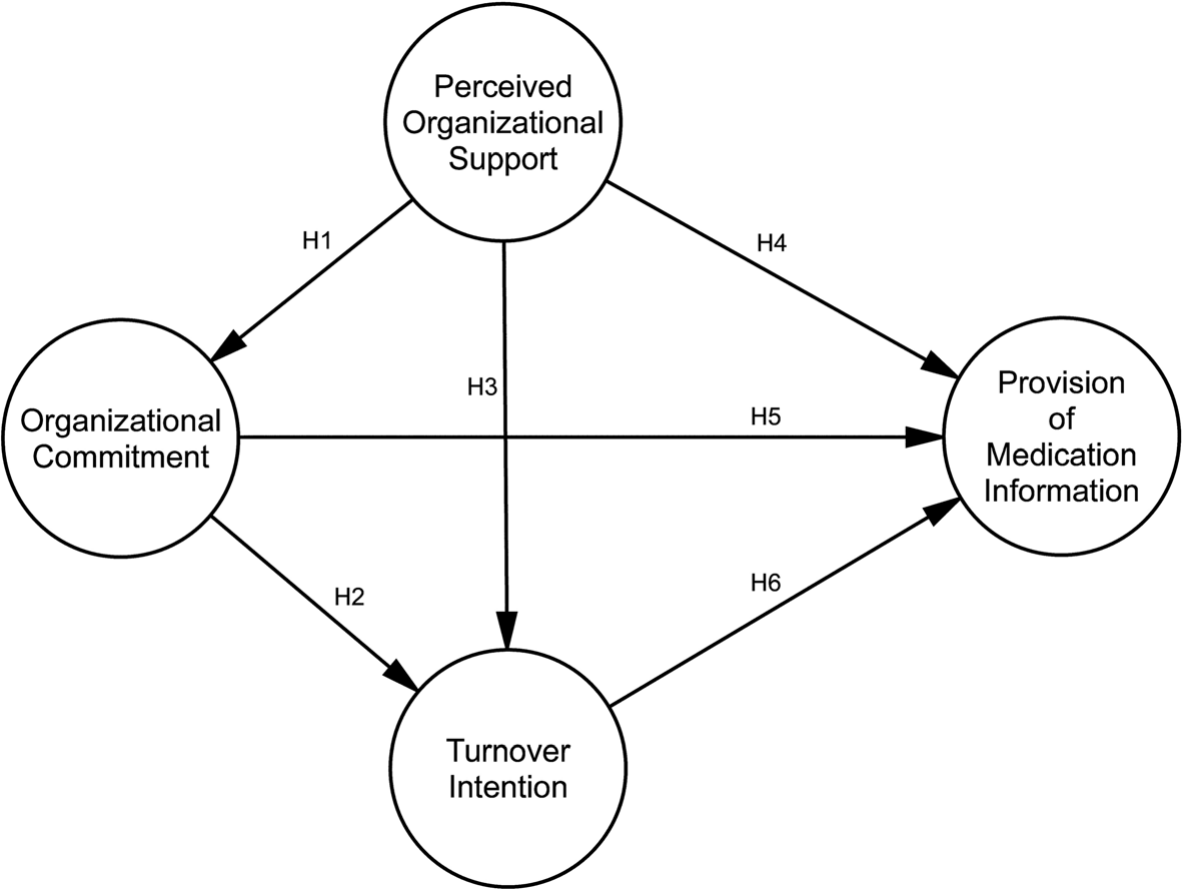
**Hypothesized model of the relationships among Perceived Organizational Support, Organizational Commitment, Turnover Intention, and Provision of Medication Information.**

A positive and patient-oriented organizational environment is especially important in community pharmacies to ensure high quality patient care due to the specific nature of community pharmacy business [32]. Usually employees who are committed to organization believe in its goals and ideals and work on behalf of the organization [24,27]. But if a pharmacist is working in a profit-oriented environment, he/she may encounter ethical dilemmas when choosing between organizational demands (e.g., profit, effectiveness) and professional obligations (e.g., quality of pharmaceutical services) [33] and may place organizational needs ahead of the needs of the patient [33,34]. The main purpose of this research was therefore to employ the structural equation modeling approach to test the hypothesized model (see Figure 1) of the relationships among the organizational variables, namely, Perceived Organizational Support, Organizational Commitment, and Turnover Intention (H1, H2, H3), and to investigate whether any of these variables affects the Provision of Medication Information at community pharmacies (H4, H5, H6).

## Methods

### Questionnaire development

Organizational support perceived by community pharmacists was estimated using the Perceived Organizational Support instrument developed by Rhoades, Eisenberger, and Armeli [35]. In our study, three questions from this questionnaire were selected to evaluate how pharmacists perceived organizational support in the pharmacies where they worked. Pharmacists’ organizational commitment was estimated by the Organizational Commitment questionnaire developed by Mowday, Steers, and Porter [25]. Four questions were chosen for this study to evaluate respondents’ commitment to their organizations. Turnover intentions were estimated by means of the Michigan Organizational Assessment Questionnaire developed by Cammann and colleagues (Cammann C, Fichman M, Jenkins D, Klesh J: The Michigan Organizational Assessment Questionnaire, 1979, unpublished). Two questions were selected to estimate pharmacists’ intentions of leaving their jobs. All the instruments were double-translated into Lithuanian and adapted to the context of the Lithuanian community pharmacy. Respondents were asked to answer to the questions according to a 5-point scale ranging from “strongly agree” to “strongly disagree.”

Also a questionnaire was developed to evaluate how community pharmacists tended to inform patients about their medicines. The questions were developed to reflect the main requirements of the service of provision of medication information as highlighted in national [36] and international standards [1-3], that is, to provide patients with clear and concise pharmaceutical information about the correct use of the drugs being dispensed. The measure was reviewed by three experts in the field of pharmacy and some questions were reformulated according to their suggestions. Respondents were asked to reflect on their actions when dealing with patients and to state how often they provided all the necessary information for patients on how to use their drugs safely, how often they warned their patients about possible drug interactions and contraindications, and how frequently they answered additional questions about the use of the drugs. Respondents were asked to answer to the questions according to a 5-point scale from “never” to “always”.

A total of 47 pharmacists agreed to test the preliminary questionnaire in a pilot study conducted in February 2012. The participants were asked to fill in the questionnaire and make some comments about whether any of the questions caused problems in understanding of the questionnaire. The respondents did not make any essential comments, although in response to their suggestions minor changes, such as wording, were made to the questionnaire. In addition, Principal Component Analysis (PCA) was conducted for each of the four measures to identify the underlying constructs, and Cronbach’s alpha values (α) were computed to estimate the internal consistency reliability.

The initial scale of Provision of Medication Information consisted of seven questions. However, two items (“How often do you inform about possible side effects of the drug being dispensed” and “How often do you explain about proper use of the drug being dispensed”) were eliminated after the PCA procedure due to low factor loadings (<0.4). The PCA of the five remaining items related to provision of medication information yielded results consistent with a one-factor solution that explained 53.09 percent of the total variance (α = 0.76) with factor loadings from 0.62 to 0.88. The Principal Component Analyses of the organizational scales resulted in single-factor solutions that accounted from 78.38 (Perceived Organizational Support) to 92.20 percent (Turnover Intention) of the total variance. The organizational scales showed very good internal consistency reliability with Cronbach’s alpha values ranging from 0.86 (Perceived Organizational Support) to 0.94 (Organizational Commitment).

### The main study

The main study was conducted in Lithuania during the period of April–June 2012. The convenience sampling method was used as the most cost-effective and time-efficient method to reach the goals of the study. The sample of the research consisted of pharmacists who were improving their qualifications at scientific-practical conferences organized by the Lithuanian University of Health Sciences in the five largest cities of Lithuania. Because each pharmacist in Lithuania is obliged to improve his/her professional qualifications by spending at least 120 h every 5 years on qualification development courses, all pharmacists had a similar probability of being included in the sample.

During the study, 420 surveys were distributed to pharmacists and 324 were returned (77.1% response rate). It is desirable in structural equation modeling to have data free of missing values [37]. For this reason, 13 surveys (4.0%) with missing answers were excluded from further analysis. After exclusion, a total of 311 surveys were selected for data analysis.

### Data analysis

The structural equation modeling analysis was performed using the statistical program AMOS 22.0. As a preliminary step in the data analysis, a confirmatory factor analysis (CFA) was performed to compare the hypothesized model against nested alternatives, consisting of three to one factors. A pairwise comparison between models was conducted by estimating the Chi-square difference (Δχ^2^) and by evaluating comparative measures of fit—Bayesian informational criterion (BIC), Akaike information criterion (AIC), Parsimony Normed Fit (PNFI), and Parsimony Comparative of Fit (PCFI) indices. Lower values of BIC and AIC and, alternatively, higher values of PNFI and PCFI would indicate a better model fit with the data.

The hypothesized model was tested for internal consistency reliability, convergent validity, and discriminant validity. Internal consistency reliability of the scales was measured using the Composite Reliability Index and the Cronbach’s alpha coefficient. The Composite Reliability Index of each of the scales was calculated according to formula suggested by Fornell and Larcker [38]: ${\rho}_{\eta }=\frac{{\left({\displaystyle \sum {\lambda}_i}\right)}^2}{{\left({\displaystyle \sum {\lambda}_i}\right)}^2+{\displaystyle \sum \left(1-{\lambda}_i^2\right)}}$ where ρ_η_—construct reliability, λ_i_—standardized loading for each observed variable. Usually values of both Cronbach’s alpha and Composite Reliability Index measures above 0.7 indicate satisfactory reliability, and values above 0.8 indicate good internal consistency reliability [39].

The convergent validity of each construct was estimated by calculating the Average Variance Extracted (AVE) coefficient according to the formula proposed by Fornell and Larcker [38]: $AVE=\frac{{\displaystyle \sum {\lambda}_i^2}}{{\displaystyle \sum {\lambda}_i^2}+{\displaystyle \sum \left(1-{\lambda}_i^2\right)}}$ where AVE—λ_i_—standardized loading for each observed variable. Bagozzi and Yi [40] recommend that AVE should be equal to or greater than 0.5.

The discriminant validity of the questionnaire was also estimated. According to Fornell and Larcker [38], discriminant validity is satisfactory if the AVE for each construct is larger than the shared variance between the constructs. The shared variance is the squared correlation between the constructs. In our study, discriminant validity was regarded as satisfactory if the square root of the AVE of each construct was lower than were the correlations among the constructs.

A second test for discriminant validity involved pairwise comparison of factors using a Chi-square difference test [41]. For each pair of factors, two models—one with unconstrained correlation and one with correlation constrained to 1.00—were developed. A significant increase in the *χ*^2^ value for the model with constrained correlation would signify discriminant validity [42].

Furthermore, to assess the potential impact of common method variance, we conducted a Harman’s single-factor test using CFA techniques [43]. A one-factor model was created in which all manifest variables were related to a single factor. The one-factor model was compared against the hypothesized four-factor model via a chi-square difference test. The Common Latent Factor technique [43] was also conducted by introducing an additional latent factor to the final model that was linked to all variables. The model was analyzed to observe the effect of common method variance. Significantly improved goodness-of-fit statistics for the one-factor model and/or the model controlled by the Common Latent Factor would signify the problem of common method bias.

The fit of the models was assessed using the following indices: Chi-Square, Normed Chi-Square (*χ*^2^/df), Adjusted Goodness-of-Fit Index (AGFI), Comparative Fit Index (CFI), Tucker-Lewis Index, Root Mean-Square Error of Approximation (RSMEA), and Standardized Root Mean Residual (SRMR). A good fit between the model and the data was supposed to exist if the p-value of the chi-square was above 0.05; Normed Chi-Square value was between 1.0 and 2.0; AGFI, CFI, and TLI values were greater than 0.95; and the RSMEA index and SRMR value were below 0.05 [44].

Because the maximum likelihood estimation method assumes that data are normally distributed, the robustness of our findings was tested further. In the case of non-normality, a bootstrapping procedure with 2000 samples drawn from the data set was applied to calculate the p-values and confidence intervals of the relationships. In addition to the traditional chi-square test, the Bollen-Stine bootstrapped version of the test was performed [45].

### Ethics and approvals

According to the national regulations of Lithuania, the ethical approval of the Lithuanian Bioethics Committee is compulsory for biomedical research [46]. Because the study was not biomedical but an organizational survey research with no vulnerable groups involved, ethical permission was not necessary. During the survey, the goals of the study were explained to respondents, and they were informed that participation was voluntary and anonymous. Filling in the questionnaire was considered to constitute informed consent.

## Results

The first step of the data analysis was to test the constructs for internal consistency reliability and convergent validity (see Table 1). All the scales showed good internal consistency reliability: the Cronbach’s alpha and Composite Reliability Index of each construct exceeded the threshold of 0.7. In addition, all the scales met the requirement of convergent validity as the AVEs of all the constructs were above the threshold of 0.5.

**Table 1:** **Descriptive statistics, internal consistency reliability, and convergent validity**

	**Mdn**	**M**	**SD**	**α**	**CR**	**AVE**
Perceived Organizational Support^a^				0.85	0.86	0.67
POS_1: The pharmacy I am working in really cares about my well-being	4	3.14	1.34			
POS_2: The pharmacy I am working in strongly considers my goals and values	4	3.27	1.33			
POS_4: The pharmacy I am working in cares about my opinions	4	3.24	1.32			
Organizational Commitment^a^				0.88	0.88	0.65
OC_2: I talk up this pharmacy to my friends as a great organization to work for	4	3.77	1.20			
OC_6: I am proud to tell others I am part of this pharmacy	4	3.35	1.26			
OC_8: This pharmacy really inspires the very best in me in terms of job performance	4	3.49	1.23			
OC_10: I am extremely glad that I chose this pharmacy to work for over others I was considering at the time I joined	4	3.36	1.29			
Turnover Intention^a^				0.86	0.86	0.76
MOAQ_1: I often think of leaving the organization	2	2.13	1.35			
MOAQ_2: It is very possible that I will look for a new job soon	1	2.11	1.34			
Provision of Medication Information^b^				0.83	0.84	0.51
PMI_1: How often do you provide all necessary information on the use of the drug being dispensed?	4	4.31	0.79			
PMI _2: How often do you inform about safe use of the drug being dispensed?	5	4.62	0.62			
PMI _3: How often do you answer all additional questions about the drug being dispensed?	5	4.52	0.66			
PMI _4: How often do you warn about the drugs that are incompatible with the drug being dispensed?	4	4.10	0.87			
PMI _5: How often do you warn about possible contraindications of the drug being dispensed?	4	3.91	0.93			

Notes: Mdn – median; M – mean; SD – standard deviation; α – Cronbach’s alpha; CR – composite reliability; AVE – average variance extracted.

^a^Five-point scale ranging from 1 (strongly disagree) to 5 (strongly agree).

^b^Five-point scale ranging from 1 (never) to 5 (always).

In addition, the constructs showed good discriminant validity; the square roots of AVE of each construct were larger than the correlations between the constructs (see Table 2). The results of the pairwise *χ*^2^ differential test (see Table 2) also showed that the discriminant validity of all the constructs was supported.

**Table 2:** **Discriminant validity**

	**Perceived organizational support**	**Organizational commitment**	**Turnover intention**	**Provision of medication information**
Perceived Organizational Support	*0.82*	169.25^*^	192.01^*^	410.89^*^
Organizational Commitment	0.73^*^	*0.81*	151.67^*^	481.83^*^
Turnover Intention	−0.54^*^	−0.66^*^	*0.87*	258.37^*^
Provision of Medication Information	0.24^*^	0.26^*^	−0.17^*^	*0.71*

* – p < 0.001.

Notes: Lower non-diagonal elements are the correlations between the constructs; diagonal elements (in italics) are the square root of the average variance extracted (AVE); upper non-diagonal elements represent chi-square difference (∆*χ* ^2^ (df = 1)) between two models—the first with correlation constrained to 1.00 and the second with unconstrained correlation.

We also conducted a CFA technique to examine the distinctiveness of the latent variables. We compared the fit of the hypothesized four-factor model with a nested alternative three-factor model (combining the dimensions of Perceived Organizational Support and Organizational Commitment), a two-factor model (combining Perceived Organizational Support, Organizational Commitment, and Turnover Intention), and a one-factor model (see Table 3). The chi-square difference test and BIC, AIC, PNFI, and PCFI coefficients indicated that the hypothesized four-factor model fitted the data significantly better than the alternative models (see Table 3). Thus, the CFA results indicated support for the construct validity of the hypothesized four-factor model.

**Table 3:** **Confirmatory factor analysis and model comparison**

**Model**	***χ*** ^**2**^ **(df)**	**∆** ***χ*** ^**2**^ **(∆df) vs hypothesized model**	**BIC**	**AIC**	**PNFI**	**PCFI**
M1: One-factor model	877.72(77)^*^	801.26(6)^*^	1038.43	933.72	0.52	0.54
M2: Two-factor model	395.94(76)^*^	319.48(5)^*^	562.39	453.94	0.69	0.71
M3: Three-factor model	245.06(74)^*^	168.6(3)^*^	422.99	307.06	0.73	0.75
M4: Four-factor (hypothesized) model	76.46(71)		271.62	144.46	0.75	0.78
M5: M4 with constrained H3, H4, and H6 (final model)	79.87(74)	3.41(3)	257.81	141.87	0.79	0.81
M6: Common Latent Factor controlled M5	79.87(73)	3.41(4)	263.54	143.87	0.77	0.80

* p < 0.001.

**Figure 2:**
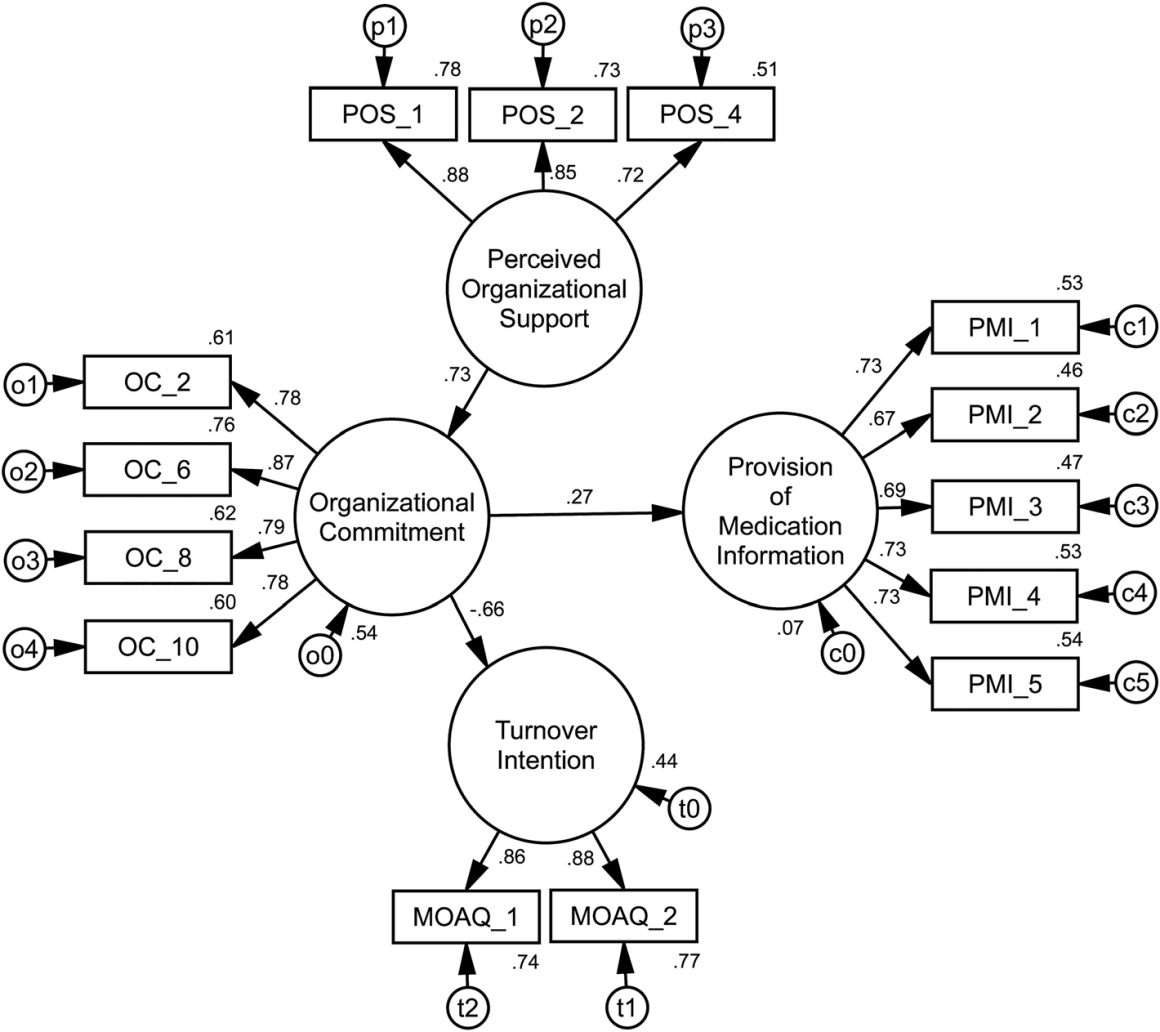
**The final model of the relationships among Perceived Organizational Support, Organizational Commitment, Turnover Intention, and Provision of Medication Information.**

The hypothesized model (M4) (see Table 3 and Figure 1) showed good fit with the data: *χ*^*2*^(71, N = 311) = 76.46, p *=* 0.31 (Normed Chi-Square = 1.08). A good-fitting model was also suggested by other fit indices: AGFI = 0.95; CFI = 1.00; TLI = 1.00; RMSEA = 0.02 [0.00; 0.04]; SRMR = 0.03. However, three paths appeared to be non-significant: from Perceived Organizational Support to Turnover Intention (H3), from Perceived Organizational Support to Provision of Medication Information (H4), and from Turnover Intention to Provision of Medication Information (H6). According to Byrne [45], non-significant parameters can be regarded as non-significant to the model and should be deleted from further analysis. After the removal of these paths (see Figure 2), the fit of the adjusted model (M5) (*χ*^*2*^(74, N = 311) = 79.88, p *=* 0.30; Normed Chi-Square = 1.08; AGFI = 0.95; CFI = 1.00; TLI = 1.00; RMSEA = 0.02 [0.00; 0.04]; SRMR = 0.04) did not worsen significantly (∆*χ*^*2*^(3) = 3.41, p = 0.33) and the reduced model was selected as the final one.

Keeping in mind that direct correlations between Perceived Organizational Support and Provision of Medication Information, as well as Turnover Intention were significant (see Table 2), also the removal of the H3 and H4 paths from the hypothesized model (M4) was not accompanied by a significant decrease in model fit and the final model (M5) had the best values for the BIC, AIC, PNFI, and PCFI coefficients when compared with the hypothesized one (see Table 3), support for full mediation was confirmed [47].

The Harman single-factor and common latent method factor tests were used to evaluate the constructs for common method bias. A single-factor model (M1) was compared to the four-factor hypothesized model (M4). The goodness-of-fit statistics of this model were significantly worse when compared to the four-factor model, indicating that the correlations between observed variables cannot be adequately explained by one factor (see Table 3). An additional model (M6) in which the final model was controlled by the Common Latent Factor was developed and compared to the final model (M5). The results showed that the chi-square statistics did not change significantly (∆*χ*^*2*^(1) = 0.00, p = 1.00), the loadings of the Common Latent Factor on all indicators were insignificant, and the effect sizes between the constructs remained unchanged. We can conclude that the relationships among the variables were not distorted by common method bias.

The data did not meet the normality criterion, and the Bollen-Stine bootstrap (2000 samples) p-value was also calculated. The value of Bollen-Stine p *=* 0.70 confirmed that the final model was consistent with the data. A bootstrap procedure was also conducted to test the significance and determine the confidence intervals of the relationships between the constructs. The results of the bootstrap procedure showed that both direct and indirect relationships between the constructs were significant (see Table 4).

**Table 4:** **Bootstrap path significance of parameter estimates (2000 samples)**

		**95% CI**	
**Path**	**Estimate**	**Lower bound**	**Upper bound**
*Standardized direct effects*			
Perceived Organizational Support → Organizational Commitment	0.73^*^	0.67	0.80
Organizational Commitment → Turnover Intention	−0.67^*^	−0.73	−0.59
Organizational Commitment → Provision of Medication Information	0.27^*^	0.16	0.37
*Standardized indirect effects*			
Perceived Organizational Support →	−0.48^*^	−0.57	−0.40
Organizational Commitment → Turnover Intention			
Perceived Organizational Support →	0.20^*^	0.10	0.30
Organizational Commitment → Provision of Medication Information			

* p < 0.001.

The final model (see Figure 2) revealed that of all the organizational variables observed in this research, Organizational Commitment alone was found to have a small but significant direct effect on Provision of Medication Information. Turnover Intention did not have a significant effect on the variable of Provision of Medication Information. However, the effects of Perceived Organizational Support were fully mediated via the variable of Organizational Commitment to both variables of Turnover Intention and Provision of Medication Information. The Organizational Commitment variable also had a negative direct effect on pharmacists’ intentions to leave their jobs.

## Discussion

The organizational variables of Perceived Organizational Support, Organizational Commitment, and Turnover Intention were found to be interrelated in different studies. Some studies [30,35,48] found that the effects of Perceived Organizational Support on Turnover Intention were fully mediated through affective commitment. Likewise, some researchers reported that normative commitment may also serve as a mediator of the relationship between Perceived Organizational Support and Turnover Intention [30,49,50]. In addition, it was proved that commitment to organizations directly and negatively affected job turnover intention [51-54]. Similarly, significant direct and indirect relationships among Perceived Organizational Support, Organizational Commitment, and Turnover Intention were observed in our study. Respondents who felt better congruence with their organizations as a result of the support they perceived as coming from their organizations had less intention of resigning from their job. Simultaneously, decreased intention to quit also was observed as the effect of increased commitment to organization. These results imply that supportive management strategies oriented to the increase of employees’ commitment may be an effective strategy to retain staff at community pharmacies.

In 2010, a study conducted in Lithuania found a significant positive relationship between pharmacy management’s policies towards quality pharmaceutical services and the quality of pharmaceutical services provided at community pharmacies [55]. The results of our study revealed that the effect of the support that pharmacists saw as coming from their organizations was fully mediated through the variable of Organizational Commitment. Respondents who felt higher levels of support from their organizations also expressed, to a certain extent, higher commitment to their organizations by providing more consistent medication information to patients. In this context, community pharmacies may be viewed as encouraging (rather indirectly) patient-oriented behavior.

Nevertheless, the extent of provision of medication information that can be predicted by organizational factors appears to be limited—only 7% in the variance of the variable of Provision of Medication Information was explicable under the model. This insight was strengthened by the fact that pharmacists’ turnover intentions, as a possible outcome of a negatively perceived organizational environment, did not significantly decrease levels of medication information provided to patients. Pharmacists remained committed to their patients and to the profession in general. This may signify some difference between the pharmacist–patient (patient-oriented) and pharmacy–patient (business-oriented) relationships [32,56,57]; pharmacists’ professional behavior may have depended more on individual factors (e.g., professional commitment, qualifications, ethical cognition) than organizational factors, something that should be explored in future studies.

The main implication following from the results is that it is not to be expected that a positive organizational environment in general will definitely result in a higher quality of pharmaceutical services. It is patient-oriented organizational policies supporting the provision of high-quality pharmaceutical services that may be seen as, to some extent, an effective way of increasing pharmacists’ congruence with their organizations as healthcare professionals and, consequently, may increase the quality of patient care at community pharmacies. Management’s support for provision of medication information at community pharmacies, therefore, may be seen as not merely benefiting patient care, but in addition increasing, to some extent, pharmacists’ organizational commitment and reducing their wishes to leave the jobs.

### Study limitations

There are some limitations to this study that can guide future research. The convenience sampling method was used for sample selection, and the results cannot be regarded as representative of the population of pharmacists in Lithuania. Although all pharmacists had an equal opportunity to participate in the scientific-practical conferences (participation was free of charge), the odds may had been distorted by different psychological, social, and other factors. A second limitation of the study is that although the relationships observed in the study were directional, they cannot be regarded as causal. Research that is more consistent should be carried out to analyze the nature of these relationships. Another limitation of the study is the use of shortened scales. Difficulties arise when comparing the results of this research with studies that used full scales. The introduction of four different scales led to the decision to shorten the scales to design a concise questionnaire and to avoid automatic filling in of the questionnaire. The scales did have adequate construct validity, however. Also, the stability of the questionnaire over time (test–retest reliability) was not examined.

## Conclusions

1. The connections among organizational variables showed signs of mediation. Support that pharmacists perceived as coming from their organizations increased their commitment to those organizations and, consequently, decreased their turnover intentions.
2. Organizational Commitment had a small but significant direct effect on Provision of Medication Information and acted as a mediator of small but significant effect of Perceived Organizational Support on Provision of Medication Information.
3. Turnover Intention was found not to have a significant effect on Provision of Medication Information.
